# (Hetero)aryl substituted thiazol-2,4-yl scaffold as human carbonic anhydrase I, II, VII and XIV activators

**DOI:** 10.1080/14756366.2018.1543292

**Published:** 2019-01-03

**Authors:** Marouan Rami, Jean-Yves Winum, Claudiu T. Supuran, Patricia Melnyk, Saïd Yous

**Affiliations:** a Université de Lille, Inserm, CHU Lille, UMR-S 1172-JPArc-Centre de Recherche Jean-Pierre AUBERT Neurosciences et Cancer, Lille59000, France;; b Institut des Biomolécules Max Mousseron (IBMM) UMR 5247 CNRS, ENSCM, Université de Montpellier, Bâtiment de Recherche Max Mousseron, Ecole Nationale Supérieure de Chimie de Montpellier, 240 avenue du Professeur Emile Jeanbrau, Montpellier Cedex34296, France;; c Neurofarba Department, Section of Pharmaceutical and Nutriceutical Sciences, Università degli Studi di Firenze, Via Ugo Schiff 6, Sesto Fiorentino, Florence50019, Italy

**Keywords:** Thiazole scaffold, carbonic anhydrase activators, isozymes, drug design, synthesis

## Abstract

Using histamine as lead molecule, a library of (hetero)aryl substituted thiazol-2,4-yl derivatives incorporating pyridine as proton shuttling moiety were obtained and investigated as activators of human carbonic anhydrase (CA, EC 4.2.1.1) isoforms I, II, VII and XIV. Some derivatives displayed good activating and selectivity profiles. This study provides an interesting opportunity to study the thiazole scaffold for the design of CA activators (CAAs), possibly acting on the central nervous system and targeting pathologies involving memory and learning impairments.

## 1. Introduction

Similar to many enzymes used as therapeutic targets, carbonic anhydrases (CAs, EC 4.2.1.1) are of particular interest considering the large spectra of physiological and pathologic processes in which they are involved. Because of their high catalytic activity, the 13 different active carbonic anhydrase isozymes can be modulated or with inhibitors, or with activators. CA inhibition has found potential in a range of therapeutic areas, and several inhibitors are exploited pharmacologically in clinic as diuretics, anticonvulsant drugs, antiglaucoma, anti-neuropathic pain agents, anti-arthritis agents, and ultimately also for the management of hypoxic tumors^1^^,^^2^.

CA activation is known more than 80 years, but pharmacological applications started to be explored in the last 15 years on diseases in which CA activity is diminished, such as aging, memory disorder, cognition impairment or Alzheimer's disease. In animal models it has been shown that CA activators are able to enhance cognition, spatial memory and learning^3^^,^^4^. In the last years, CAAs drug design studies afforded a large number of potent but nonselective CA activators. Natural and non-natural amino-acids as well as aromatic/heterocyclic amines were among the first activators described in literature. First X-ray crystal structure of histamine as hCA II activator was reported in 1997^5^.Several other X-ray structures with amines or amino-acids activators were published among which l- and d-His, l- and d-Phe, d-Trp and l-adrenaline^4^.

**Figure 1.**
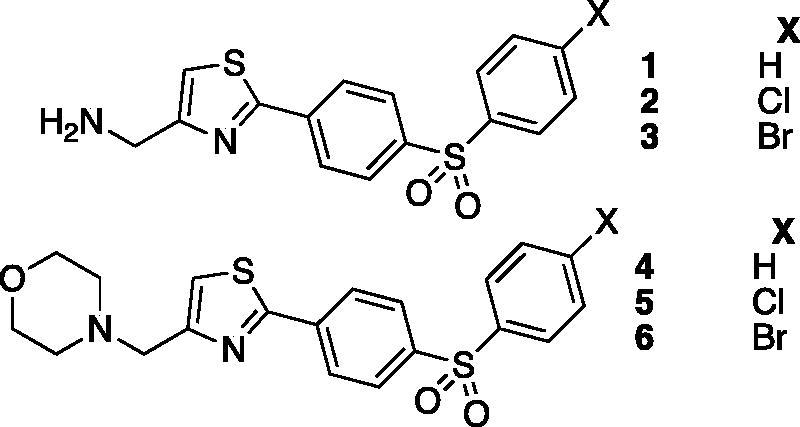
Thiazole activators reported in Ref. (14).

One of the main drawback with the activator of amine and amino-acid types is their lack of selectivity for various CA isoforms. Based on this fact, derivatized histamine^6^^,^^7^, substituted histidine^6^^,^^7^ and *bis*-imidazoles compounds^8^ were reported as CA activators. Heterocyclic scaffolds were also studied such as piperazine^9^, diketopiperazine^10^, indazole, pyrazole, oxazole^11^, triazole^12^ and thiadiazole^13^, leading to active carbonic anhydrase activators with enhanced selectivity. The thiazole scaffold is a privileged one in drug discovery, but very few CA activators incorporating this heterocyclic scaffold were reported so far. In 2001, thiazole activators compounds **1**, **2** and **3** depicted in Figure 1 were reported as micromolar CA activators against hCA I and hCA II, whereas compounds **4**, **5** and **6** were found to be inactive^14^.

In order to expand the scope of using thiazole scaffold in the design of CA activators, we present here the synthesis and CA activation studies of a library of derivatives having (hetero)aryl substituted thiazol-2,4-yl scaffold and the pyridine group as proton shuttling moiety, which have been investigated on a panel of selected CA isozymes involved in brain function.

## 2. Materials and methods

### 2.1. Chemistry

#### General

All common reagents and solvents were obtained from commercial sources (Sigma-Aldrich Alfa Aesar or Acros organics) and used without further purification. Compounds were purified on a glass column using Merck Silica Gel 60 (230–400 mesh). Their purity and Mass spectra were determined on Surveyor MSQ Thermoelectron spectrometer (+cAPCI corona sid =30.00, det =1400.00 Full ms [100.00–1000.00]). Melting points were determined with a büchi 510 capillary apparatus and are uncorrected. ^1^H NMR spectra were recorded on a Bruker AC400P spectrometer using DMSO-*d_6_* or CDCl_3_ as solvents. Chemical shifts are reported in δ units (parts per million) relative to (Me)_4_Si as internal standard, and coupling constants (*J*) are expressed in Hertz. Infrared spectra were obtained on a Perkin–Elmer FT-IR S1000 on KBr paths.

#### General procedure for the synthesis of thiazol-4-yl-methanamine arylsubstituted (13a-f) and thiazol-4-yl-ethanamine arylsubstituted (10a-c) and pyridinyl thiazol-4-yl acetamide (16a-b)

To a solution of acid (1.1 mmol) in dichloromethane (20 ml) was added triethylamine (1.2 mmol) at 0 °C. Hydroxybenzotriazole (1.2 mmol) and 1-Ethyl-3-(3-dimethylaminopropyl)carbodiimide hydrochloride (1.2 mmol) were added to a solution. The reaction mixture was stirred at 0 °C 30 min. Corresponding primary amine (1 mmol) was added to a solution. The reaction was stirred at 0 °C for 1 h and at room temperature for 1 h. The reaction mixture was hydrolyzed (H_2_O) and extracted with dichloromethane. The organic layer was dried (MgSO_4_), filtered, and concentrated under reduced pressure. The crude was purified by column chromatography (SiO_2_), or recrystallized from appropriate solvent.

#### N-{2-[4–(3-Chloro-phenyl)-thiazol-2-yl]-ethyl}-nicotinamide (10a)

White solid; Yield: 60%; mp: 91–93 °C; ^1^H NMR (400 MHz, DMSO-*d_6_*) δ ppm 3.31 (m, 2H), 3.70 (m, 2H), 7.38 (m, 1H), 7.48 (m, 2H), 7.89 (td, *J* = 7.57 Hz, *J* = 1.45 Hz, 1H), 7.99 (t, *J* = 1.61 Hz, 1H), 8.15 (m, 2H), 8.70 (dd, *J* = 4.95 Hz, *J* = 1.74 Hz, 1H), 8.89 (t, *J* = 5.53 Hz, 1H), 8.87 (dd, *J* = 2.18 Hz, *J* = 0.72 Hz, 1H); ^13^^ ^C NMR (101 MHz, DMSO-*d_6_*) δ ppm 32.41, 38.59, 115.41, 123.38, 124.43, 125.49, 127.57, 129.77, 130.58, 133.51, 134.78, 136.05, 148.20, 151.80, 152.12, 164.85, 167.71; IR (neat cm^−1^) 1634 (CO), 3306 (NH); *m/z* 344.49 [*M*+*H*]^+^.

#### N-{2-[4–(3-Chloro-phenyl)-thiazol-2-yl]-ethyl}-isonicotinamide (10b)

White solid; Yield: 68%; mp: 156–158 °C; ^1^H NMR (400 MHz, CDCl_3_) *δ* 3.35 (t, *J* = 5.76 Hz, 2H), 3.96 (m, 2H), 7.36 (m, 2H), 7.44 (m, 1H), 7.71 (m, 3H), 7.92 (m, 2H), 8.77 (brs, 2H); ^13^^ ^C NMR (101 MHz, DMSO-*d_6_*) δ ppm 31.94, 38.15, 117,49, 123.22, 128.89, 133.35, 134.80, 136.09, 146.66, 147.27, 149.81, 150.66, 152.09, 163.19, 168.54; IR (neat cm^−1^) 1637 (CO), 3271 (NH); LC/MS *m/z* 344.50 [*M*+*H*]^+^.

#### N-{2-[4–(3-Chloro-phenyl)-thiazol-2-yl]-ethyl}-2-pyridin-4-yl-acetamide (10c)

White solid; Yield: 67%; mp: 123–125 °C; ^1^H NMR (400 MHz, CDCl_3_) δ 3.23 (t, *J* =  6.02 Hz, 2H), 3.55 (s, 2H), 3.75 (m, 2H), 6.44 (brs, 1H), 7.18 (m, 2H), 7.23 (m, 3H), 7.65 (m, 1H), 8.47 (m, 1H), 8.47 (brs, 2H); ^13^^ ^C NMR (101 MHz, DMSO-*d_6_*) δ ppm 26.95, 32.87 42.14, 116.12, 122.21, 127.87, 131.32, 131.78, 135.12, 144.56, 146.28, 148.52, 149.21, 151.11, 162.26, 167.61; IR (neat cm^−1^) 1639 (CO), 3300 (NH); LC/MS *m/z* 358.59 [*M*+*H*]^+^.

#### N-[(4–(2-Chloro-phenyl)-thiazol-2-yl)methyl]-isonicotinamide (13a)

White solid; Yield: 70%; mp: 121–123 °C. ^1^H NMR (400 MHz, DMSO-*d_6_*) δ ppm 4.83 (d, *J* = 5.67 Hz, 2H), 7.41 (m, 2H), 7.55 (dd, *J* = 7.46 Hz, *J* = 1.89 Hz, 1H), 7.82 (dd, *J* = 4.51 Hz, *J* = 1.45 Hz, 2H), 7.87 (dd, *J* = 7.42 Hz, *J* = 2.03 Hz, 1H), 8.01 (brs, 1H), 8.77 (dd, *J* = 4.51 Hz, 1.45 Hz, 2H), 9.76 (t, *J* = 5.82 Hz, 1H); ^13^^ ^C NMR (101 MHz, DMSO-*d_6_*) δ ppm 41.01, 119.16, 121.15, 127.28, 129.46, 130.27, 130.82, 132.31, 132.66, 140.47, 150.18, 150.35, 165.03, 167.95; IR (neat cm^−1^) 1639 (CO), 3274 (NH); LC/MS *m/z* 330.79 [*M*+*H*]^+^.

#### N-[(4–(4-Chloro-phenyl)-thiazol-2-yl)methyl]-nicotinamide (13b)

White solid; Yield: 64%; mp: 173–175 °C; ^1^H NMR (400 MHz, DMSO-*d_6_*) δ ppm 4.81 (d, 6.11 Hz, 2H), 7.48 (m, 1H), 7.51 (m, 1H), 7.55 (ddd, *J* = 8.00 Hz, *J* = 4.95 Hz, *J* = 0.72 Hz, 1H), 7.97 (m, 2H), 8.09 (brs, 1H), 8.24 (m, 1H), 8.74 (dd, *J* = 4.80 Hz, 1.74 Hz, 1H), 9.06 (dd, *J* = 2.33 Hz, *J* = 0.72 Hz, 1H), 9.66 (t, *J* = 5.82 Hz, 1H); ^13^^ ^C NMR (101 MHz, DMSO-*d_6_*) δ ppm 41.03, 115.05, 123.55, 127.55, 128.74, 129.09, 132.39, 132.82, 135.82, 135.00, 148.34, 152.34, 152.22, 152.45, 165.11, 169.52; IR (neat cm^−1^) 1631 (CO), 3242 (NH); LC/MS *m/z* 330.71 [*M*+*H*]^+^.

#### N-[(4–(3-Chloro-phenyl)-thiazol-2-yl)methyl]-nicotinamide (13c)

White solid; Yield: 77%; mp: 164–166 °C; ^1^H NMR (400 MHz, DMSO-*d_6_*) δ ppm 4.83 (d, *J* = 5.82 Hz, 2H), 7.39 (m, 1H), 7.47 (t, *J* = 7.86 Hz, 1H), 7.55 (ddd, *J* = 8.00 Hz, *J* = 4.80 Hz, *J* = 0.87 Hz, 1H), 7.92 (td, *J* = 7.71 Hz, 1.31 Hz, 1H), 8.01 (t, *J* = 1.74 Hz, 1H), 8.19 (brs, 1H), 8.26 (m, 1H), 8.74 (dd, *J* = 5.10 Hz, 1.45 Hz, 1H), 9.08 (dd, *J* = 2.33 Hz, *J* = 0.72 Hz, 1H), 9.67 (t, *J* = 5.97 Hz, 1H); ^13^^ ^C NMR (101 MHz, DMSO-*d_6_*) δ ppm 41.04, 115.82, 123.53, 124.39, 125.47, 127.66, 129.09, 130.64, 133.56, 135.00, 135.95, 148.36, 152.07, 152.21, 165.15, 169.57; IR (neat cm^−1^) 1634 (CO), 3290 (NH); LC/MS *m/z* 330.20 [*M*+*H*]^+^.

#### N-[(4–(3-Chloro-phenyl)-thiazol-2-yl)methyl]-isonicotinamide (13d)

White solid; yield: 69%; mp: 132–134 °C; ^1^H NMR (400 MHz, DMSO-*d_6_*) *δ* 4.83 (d, *J* = 5.97 Hz, 2H), 7.41 (m, 2H), 7.50 (dd, *J* = 7.42 Hz, *J* = 1.74 Hz, 1H), 7.82 (dd, *J* = 4.51 Hz, *J* = 1.45 Hz, 2H), 7.92 (dd, *J* = 7.42 Hz, *J* = 2.02 Hz, 1H), 8.01 (s, 1H), 8.77 (dd, *J* = 4.51 Hz, *J* = 1.45 Hz, 2H), 9.77 (t, *J* = 5.82 Hz, 1H); ^13^^ ^C NMR (101 MHz, DMSO-*d_6_*) δ ppm 41.59, 119.74, 121.72, 127.86, 136.04, 130.84, 131.39, 131.88, 133.24, 141.09, 150.76, 150.92, 165.61, 168.53; IR (neat cm^−1^) 1633 (CO), 3248 (NH); LC/MS *m/z* 330.19 [*M*+*H*]^+^.

#### N-[(4–(2-Methoxy-phenyl)-thiazol-2-yl)methyl]-isonicotinamide (13e)

White solid; Yield: 81%; mp: 138–140 °C; ^1^H NMR (400 MHz, DMSO-*d_6_*) δ ppm 3.91 (s, 3H), 4.82 (d, *J* = 5.82 Hz, 2H), 7.04 (m, 1H), 7.13 (d, *J* = 7.71 Hz, 1H), 7.33 (m, 1H), 7.82 (dd, *J* = 4.51 Hz, *J* = 1.47 Hz, 2H), 8.00 (brs, 1H), 8.14 (dd, *J* = 7.86 Hz, *J* = 1.74 Hz, 2H), 8.77 (dd, *J* = 4.36 Hz, *J* = 1.74 Hz, 1H), 9.74 (t, *J* = 6.11 Hz, 1H); ^13^^ ^C NMR (101 MHz, DMSO-*d_6_*) δ ppm 55.41, 111.63, 117.63, 120.42, 121.14, 122.24, 128.96, 130.19, 132.21, 129.14, 140.52, 149.57, 150.35, 156.38, 164.38, 164.97, 166.93; IR (neat cm^−1^) 1643 (CO), 3303 (NH); LC/MS *m/z* 326.13 [*M*+*H*]^+^.

#### N-[(4–(2-Methoxy-phenyl)-thiazol-2-yl)methyl]-3-pyridin-3-yl-propionamide (13f)

White solid; Yield: 59%; mp: 200–202 °C; ^1^H NMR (400 MHz, DMSO-*d_6_*) δ ppm 2.67 (t, *J* = 6.98 Hz, 2H), 3.09 (t, *J* = 7.13 Hz, 2H), 3.91 (s, 3H), 4.54 (d, *J* = 6.11 Hz, 2H), 7.01 (m, 1H), 7.12 (m, 1H), 7.30 (m, 1H), 7.93 (brs, 1H), 7.95 (m, 1H), 8.09 (dd, *J* = 7.71 Hz, *J* = 1.74 Hz, 1H), 8.53 (dt, 8.15 Hz, 1.6 Hz, 1H), 8.77 (m, 1H), 8.87 (m, 1H), 8.99 (t, *J* = 6.97 Hz, *J* = 5.82 Hz, 1H); ^13^^ ^C NMR (101 MHz, DMSO-*d_6_*) δ ppm 27.41, 34.96, 55.44, 111.64, 117.45, 120.42, 122.21, 126.78, 128.96, 129.14, 139.03, 140.87, 141.23, 146.25, 149.44, 156.36, 167.63, 170.96); IR (neat cm^−1^) 1657 (CO), 3329 (NH); LC/MS *m/z* 354.23 [*M*+*H*]^+^.

#### N-(3-Methoxy-benzyl)-2–(2-pyridin-3-yl-thiazol-4-yl)-acetamide (16a)

White solid; Yield: 68%; mp: 110–112 °C; ^1^H NMR (400 MHz, DMSO-*d_6_*) δ ppm 3.67 (s, 3H), 3.75 (s, 2H), 4.29 (d, *J* = 5.97 Hz, 2H), 6.82 (m, 3H), 7.21 (t, *J* = 7.86 Hz, 1H), 7.55 (ddd, *J* = 8.00 Hz, *J* = 4.8 Hz, *J* = 0.72 Hz, 1H), 7.58 (brs, 1H), 8.27 (m, 1H), 8.58 (t, *J* = 5.97 Hz, 1H), 8.66 (dd, *J* = 4.8 Hz, *J* = 1.45 Hz, 1H), 9.11 (dd, *J* = 2.30 Hz, *J* = 0.72 Hz, 1H); ^13^^ ^C NMR (101 MHz, DMSO-*d_6_*) δ ppm 38.18, 42.01, 54.84, 111.91, 112.69, 117.68, 119.16, 124.12, 128.89, 129.18, 133.36, 140.36, 140.92, 146.69, 150.70, 152.13, 159.21, 168.61; IR (neat cm^−1^) 1642 (CO), 3292 (NH); LC/MS *m/z* 340.23 [*M*+*H*]^+^.

#### N-[2-Pyridin-3-yl)ethyl)-2–(2-(pyridin-3-yl)thiazol-4-yl]acetamide (16b)

White solid; Yield: 75%; mp: 117–119 °C; ^1^H NMR (400 MHz, DMSO-*d_6_*) δ ppm 2.24 (s, 2H), 2.50 (t, *J* = 6.83 Hz, 2H), 3.09 (m, 2H), 7.01 (m, 1H), 7.30 (m, 3H), 7.91 (m, 1H), 7.98 (m, 1H), 8.15 (m, 2H), 8.39 (m, 1H), 8.83 (d, *J* = 1.6 Hz, 1H); ^13^^ ^C NMR (101 MHz, DMSO-*d_6_*) δ ppm 31.94, 38.15, 40.26, 117.49, 123.22, 124.11, 128.89, 133.35, 134.80, 136.09, 146.66, 147.28, 149.81, 150.66, 152.09, 163.19, 168.54; IR (neat cm^−1^) 1635 (CO), 3282 (NH); LC/MS *m/z* 325.0 [*M*+*H*]^+^.

### 2.2. Carbonic anhydrase assays

A stopped-flow method^15^ has been used for assaying the CA catalyzed CO_2_ hydration activity with Phenol red as indicator, working at the absorbance maximum of 557 nm, following the initial rates of the CA-catalyzed CO_2_ hydration reaction for 10–100 s. For each activator, at least six traces of the initial 5–10% of the reaction have been used for determining the initial velocity. The uncatalyzed rates were determined in the same manner and subtracted from the total observed rates. Stock solutions of activator (0.1 mM) were prepared in distilled-deionized water and dilutions up to 0.1 nM were done thereafter with the assay buffer. The activation constant (*K*_A_), defined similarly with the inhibition constant *K*_I_, was obtained by considering the classical Michaelis–Menten equation (Equation 1), which has been fitted by nonlinear least squares by using PRISM 3:

(1) $$v=v_{\text{max}}/\{1+K_{M}/[S]\;(1+{\left[A\right]}_{f}/K_{A}\left.\;\right)\left.\right\}$$

where [*A*]_f_ is the free concentration of activator.

Working at substrate concentrations considerably lower than *K*_M_ ([*S*]≪*K*_M_), and considering that [*A*]_f_ can be represented in the form of the total concentration of the enzyme ([*E*]_t_) and activator ([*A*]_t_), the obtained competitive steady-state equation for determining the activation constant is given by Equation 2:

(2) $$v=v_{0}K_{A}/\{K_{A}+\left({\left[A\right]}_{t}\right.-0.5\left\{\left({\left[A\right]}_{t}\right.\right.+{\left[E\right]}_{t}+\;K_{A}\left.\right)\;-\;{\left\{\left({\left[A\right]}_{t}\right.\right.+{\left[E\right]}_{t}+\;K_{A}\left.\right)}^{2}\;-\;4{\left[A\right]}_{t}{\left[E\right]}_{t}^{1/2}\left.\right\}\}$$

where *v*_0_ represents the initial velocity of the enzyme-catalyzed reaction in the absence of an activator. All CA isozymes used in the experiments were purified recombinant proteins obtained as reported earlier by our group^6^^,^^16–23^.

## 3. Results and discussion

### 3.1. Chemistry

The small library of (Hetero)aryl substituted thiazol-2,4-yl derivatives was synthesized as follows, obviously considering histamine, a well investigated CA activator^5^, as lead molecule. The drug design rationale was to use the substituted thiazole-aminoethyl/aminomethyl scaffold known to possess affinity for the CA active site, by introducing a diverse proton-shuttling moiety (PSM) of the pyridine type, in order to generate new CA activators. Pyridine-carboxylic acids and pyridine-acetic acids were used to introduce this less investigated PSM in the molecules of the new CA activators reported here, as shown in Schemes 1–3.

**Figure:**
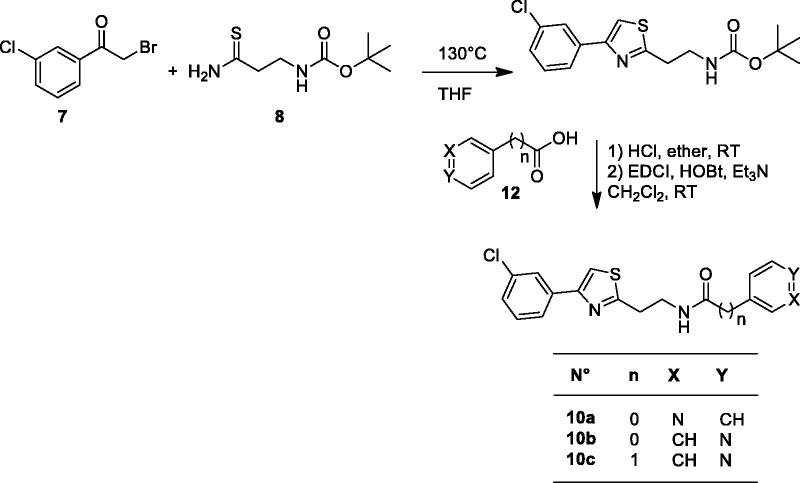
**Scheme 1**. Synthesis of thiazoles **10a–c.**

**Figure:**
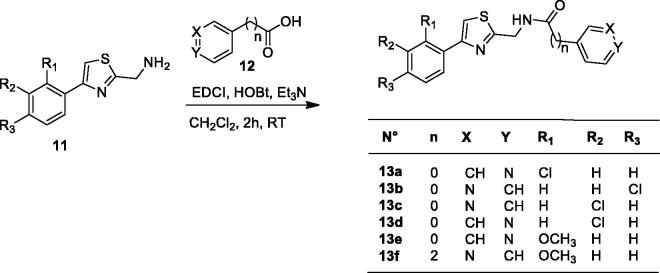
**Scheme 2**. Synthesis of thiazoles **13a–f.**

**Figure:**
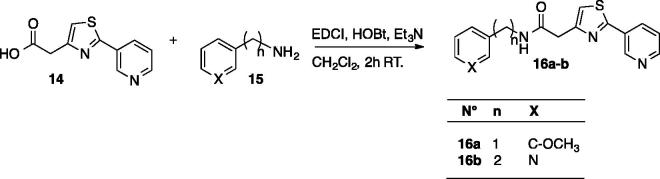
**Scheme 3**. Synthesis of thiazoles **16a–b.**

To access compounds **10a–c**, we used a strategy depicted in Scheme 1, using a three steps procedure: (i) condensation between *tert*-butyl *N*-(3-amino-3-thioxopropyl)carbamate **8** and 3-chlorophenacylbromide **7** commercially available in THF. (ii) The obtained carbamate was converted to the corresponding amine dihydrochloride by treatment with HCl (gas) at room temperature, (iii) coupling of the primary amine with the corresponding carboxylic acid to lead to target compounds **10a–c** (Scheme 1).

Coupling between carboxylic acid **12** and the 4-arylthiazol-2-yl methamine in dichloromethane using EDCI as a coupling reagent, with HOBt and triethylamine as a base, led to derivatives **13a–f** as illustrated in Scheme 2.

Compounds **16a–b** were prepared using the same simple strategy by coupling the 2-(pyridin-3-yl)-thiazol-4-yl acetic acid **14** with amine **15**.

All final derivatives were obtained in a good yield (59–81%) after purification by column chromatography (SiO_2_), or after recrystallization from appropriate solvent.

### 3.2. CA activation

Activation data against four physiologically relevant hCA isoforms, hCA I, II, VII and XIV, are shown in Table 1 using histamine as standard activator.

**Table 1.** CA activation of isoforms hCA I, II, and VII (cytosolic) and XIV (membrane-associated) with compounds **10a–c**, **13a–f**, and **16a–b** by a stopped-flow CO_2_ hydrase assay.

		*K*_A_ (μM)*		
Compound	hCA I	hCA II	hCAVII	hCA XIV
Histamine	2.1	125	37.5	0.010
**10a**	38.7	69.3	82.1	27.1
**10b**	21.6	84.9	91.0	40.3
**10c**	44.8	115.6	140.2	65.4
**13a**	13.7	74.3	64.6	31.6
**13b**	38.5	68.9	44.7	28.4
**13c**	29.1	112.4	73.8	30.9
**13d**	12.2	75.1	97.9	46.5
**13e**	6.0	98.7	66.8	25.4
**13f**	10.4	76.9	132.4	78.8
**16a**	63.4	68.1	7.5	28.7
**16b**	9.2	70.4	45.8	18.3

* Mean from three different assays (errors in the range of ±5–10% of the reported values, data not shown).

All the derivatives tested were active in the nanomolar range against the different isoforms tested.

The structure-activity relationship (SAR) is not easy to rationalize for each isoform. However, the following should be noted:

- Isoform hCA I was the one which was most activated by the new compounds reported here, which showed KAs in the range of 6.0–63.4 µM (Table 1). The best activators, among which 13e, 13f and 16b incorporated either the isonicotinoyl or the nicotinoyl moieties as PSM, and the methoxy group on the phenyl, which when absent, led to activators with much less effective properties. Compound 16b on the other hand incorporates two PSMs of the 3-pyridyl type, whereas the structurally related derivative with only one such functionality, was 6.5 times less effective as hCA I activator. All compounds investigated here were less effective than histamine (standard derivative)^5^ as activators.
- Isoform hCA II, which is normally less sensitive to amine activators^4^,^5^, was moderately activated by all compounds investigated here, which showed KAs in the range of 68.1–115.6 µM (Table 1). Thus, they were more effective activators compared to histamine, but the activation was not highly significant in these high micromolar concentration ranges. Furthermore, the SAR is not very conclusive, since most of the derivatives showed a rather similar behavior, irrespective of the substitution pattern and the PSM present in their molecule.
- The brain-associated cytosolic isoform CA VII showed a rather similar behavior to what mention above for CA II, except for 16a, which was an effective activator with a KA of 7.5 µM (5 times a better activator than histamine, Table 1). This is the only compound incorporating one 3-pyridyl PSM and the 3-methoxy-phenylmethyl tail, among the investigated activators. The structurally related compound with a second 3-pyridyl moiety 16b, was around 9 times less effective as CA VII activator compared to 16a, proving that small structural changes are essential for the biological activity. In addition, 16a is also a CA VII-selective activator, since its affinity for the other investigated isoforms is in the range of 28.7–68.1 µM.
- The transmembrane isoform hCA XIV, also present in the brain as well as in other tissues, was moderately activated by the investigated derivatives, which showed KAs in the range of 18.3–78.8 µM. Again the SAR is rather difficult to delineate since most compounds showed similar and modest activating properties.

## 4. Conclusion

In this paper, we report the synthesis of 2-(hetero)aryl thiazole and 4-(hetero)aryl thiazole compounds acting as activators of the hCAs (CAAs), obtained considering histamine as lead. Both the imidazole and the aminoethyl parts of the lead were extensively changed, replacing the first with a thiazole scaffold and extensively derivatizing the aliphatic amine part of the molecules, by introducing pyridyl type of PSMs (3- and 4-pyridyl). This new family of CAAs derivatives could be of interest in the drug design of agent acting on the central nervous system affecting pathologies mainly characterized from memory and learning impairments, considering the fact that some of them were low micromolar activators of CA I and VII, and one compound also showed CA VII-selective activator profile.
